# Supplementary material for: Electro-Design of Bimetallic PdTe Electrocatalyst for Ethanol Oxidation: Combined Experimental Approach and Ab Initio Density Functional Theory (DFT)—Based Study
Source: Nanomaterials (Basel). 2022 Oct 14;12(20):3607. doi: 10.3390/nano12203607 (PMC9610566; doi:10.3390/nano12203607)
Supplement: Supplementary file 1 [file nanomaterials-12-03607-s001.zip › nanomaterials-1947522-supplementary conversion.pdf]

nanomaterials

MDPI

Supporting Information

Electro-Design of Bimetallic PdTe Electrocatalyst for Ethanol Oxidation: Combined Experimental Approach and Ab Initio Density Functional Theory (DFT)-Based Study

Andile. Mkhohlakali [1,5,*], Xolile Fuku [2], Min Ho Seo [3], Mmalewane Modibedi [4], Lindiwe Khotseng [5] and Mkhulu Mathe [6]

1 Analytical Chemistry Division, Mintek, 200 Malibongwe Drive, Randburg 2194, South Africa
2 Institute of Nanotechnology and Water Sustainability, College of Science, Engineering and Technology, University of South Africa, Florida Science Campus, Roodepoort 1710, South Africa
3 Department of Nanotechnology Engineering, Pukyong National University, 45 Yongso-ro, Nam-gu, Busan 48547, South Korea
4 Council for Scientific and Industrial Research (CSIR), Energy Center, Pretoria 0012, South Africa
5 Department of Chemistry, University of the Western Cape, Private Bag X17, Bellville, Cape Town 7535, South Africa
6 Department of Chemistry, ICES, CSET, University of South Africa, Florida Science Campus, Roodepoort 1710, South Africa
* Correspondence: andilem@mintek.co.za

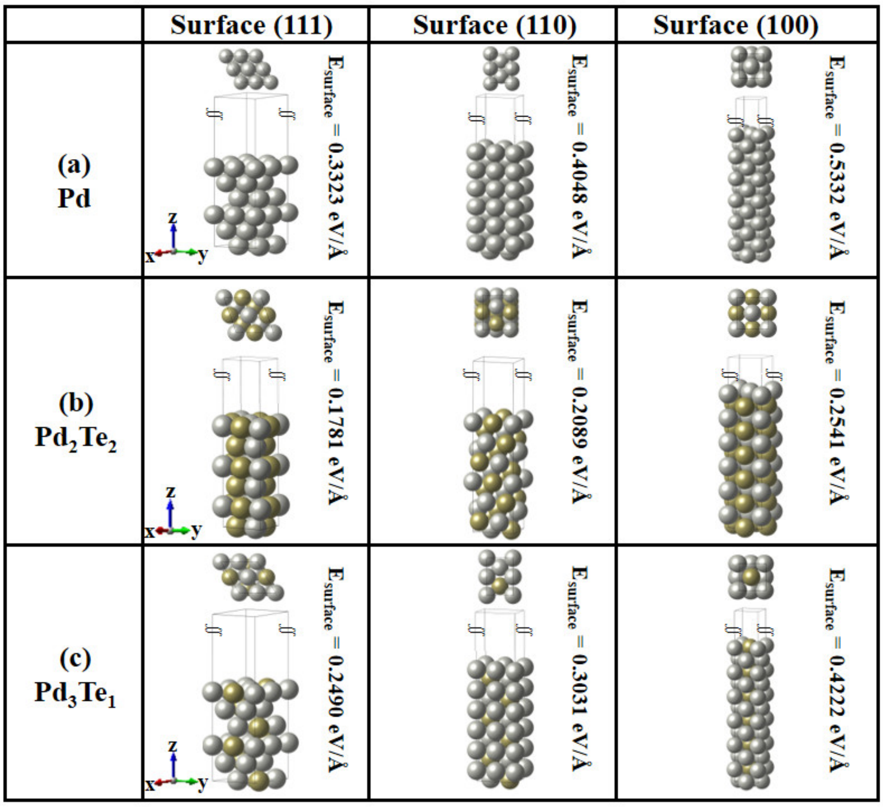

Figure S1. Defined slab models on oxygen adsorbed Pd_xTe_y (111), (110), (100) designed from bulk structure to find out which structure is the most stable surface. The three layers below were fixed to simulate surface, and identical 24 atoms were used to describe the same condition.

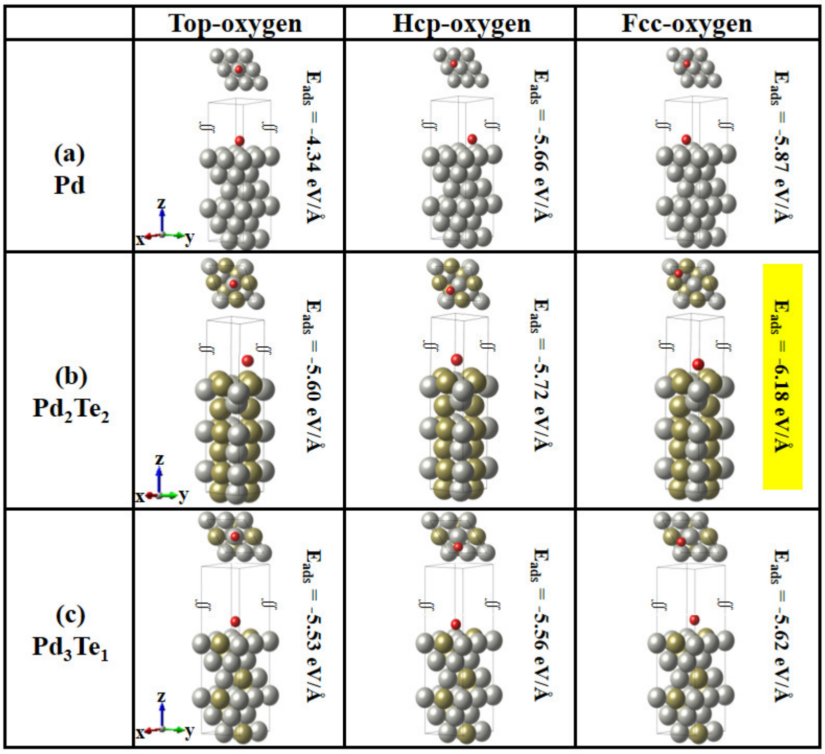

Figure S2. Defined slab models on oxygen adsorbed Pd_xTe_y (111) designed from fcc bulk structure for calculating oxygen adsorption energies. The three layers below were fixed to simulate surface, and identical 24 atoms were used to describe the same condition.

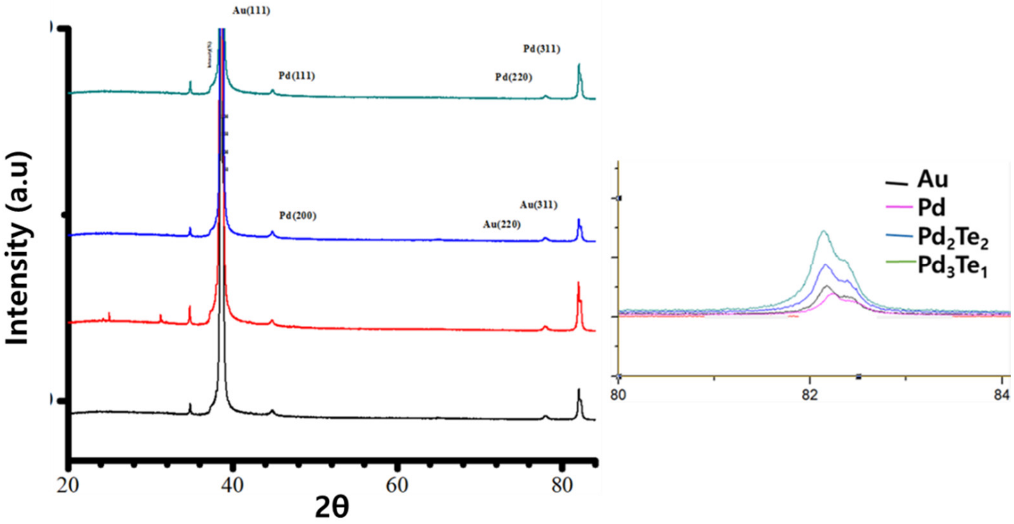

Figure S3. Experimentally XRD pattern of Au, Pd , Pd_3Te_1 and Pd_2Te_2.

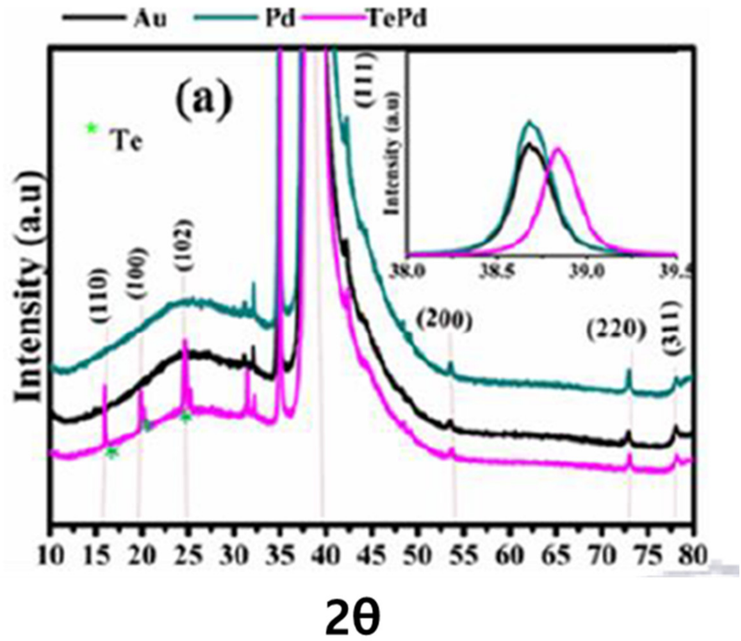

Figure S4. Experimental XRD pattern of Pd and PdTe nano film, and insert: zoomed Pd/Au (111).

Equation (S1);

$$Cu^{2+} + 2e^{-} \rightarrow Cu(s) \quad (1)$$

The deposition of a Cu-UPD, which occurs positive to Nernst positive potential as expressed in Equation (S2). While OPD Equation (S3) occurs past Nernst equilibrium potential as depicted in the following equations;

$$Cu^{2+} + 2e^{-} \leftrightarrow Cu_{(UPD)} \quad (S2)$$

$$Cu^{2+} + 2e^{-} \leftrightarrow Cu_{(OPD)} \quad (S3)$$

$$Pd + OH^{-} \leftrightarrow Pd\text{–}OH_{ads} + e^{-} \quad (S8)$$

$$Pd + CH_3CH_2OH \leftrightarrow Pd\text{–}(CH_3CH_2OH)_{ads} \quad (S9)$$

$$Pd\text{–}(CH_3CH_2OH)_{ads} + 3OH^{-} \rightarrow Pd\text{–}(CH_3CO)_{ads} + 3H_2O + 3e^{-} \quad (S10)$$

$$Pd\text{–}(CH_3CO)_{ads} + Pd - 3OH_{ads} \rightarrow Pd\text{–}CH_3COOH + Pd \quad (S11)$$

$$Pd\text{–}CH_3COOH + OH^{-} \rightarrow Pd + CH_3COO^{-} + H_2O \quad (S12)$$
